# Are endemic species necessarily ecological specialists? Functional variability and niche differentiation of two threatened *Dianthus* species in the montane steppes of northeastern Iran

**DOI:** 10.1038/s41598-020-68618-7

**Published:** 2020-07-16

**Authors:** Maryam Behroozian, Hamid Ejtehadi, Farshid Memariani, Simon Pierce, Mansour Mesdaghi

**Affiliations:** 1 Quantitative Plant Ecology and Biodiversity Research Laboratory, Department of Biology, Faculty of Science, Ferdowsi University of Mashhad, Mashhad, Iran; 2 Department of Botany, Research Center for Plant Science, Ferdowsi University of Mashhad, Mashhad, Iran; 3 Department of Agricultural and Environmental Sciences (DiSAA), University of Milan, Via G. Celoria 2, 20133 Milan, Italy; 4 Department of Range and Watershed Management, Faculty of Natural Resources and Environment, Ferdowsi University of Mashhad, Mashhad, Iran

**Keywords:** Community ecology, Conservation biology

## Abstract

Endemic species are believed to converge on narrow ranges of traits, with rarity reflecting adaptation to specific environmental regimes. We hypothesized that endemism is characterized by limited trait variability and environmental tolerances in two *Dianthus* species (*Dianthus pseudocrinitus* and *Dianthus polylepis*) endemic to the montane steppes of northeastern Iran. We measured leaf functional traits and calculated Grime’s competitor/stress-tolerator/ruderal (CSR) adaptive strategies for these and co-occurring species in seventy-five 25-m^2^ quadrats at 15 sites, also measuring a range of edaphic, climatic, and topographic parameters. While plant communities converged on the stress-tolerator strategy, *D. pseudocrinitus* exhibited functional divergence from S- to R-selected (C:S:R = 12.0:7.2:80.8% to 6.8:82.3:10.9%). Canonical correspondence analysis, in concert with Pearson’s correlation coefficients, suggested the strongest associations with elevation, annual temperature, precipitation seasonality, and soil fertility. Indeed, variance (*s*^2^) in R- and S-values for *D. pseudocrinitus* at two sites was exceptionally high, refuting the hypothesis of rarity via specialization. Rarity, in this case, is probably related to recent speciation by polyploidy (neoendemism) and dispersal limitation. *Dianthus polylepis*, in contrast, converged towards stress-tolerance. ‘Endemism’ is not synonymous with ‘incapable’, and polyploid neoendemics promise to be particularly responsive to conservation.

## Introduction

Endemic species (taxa unique to a defined geographic area) are characteristic elements of local biodiversity. Narrow endemics occupy distinct habitats, often associated with restricted ranges of environmental conditions^1,2^, or small overall geographic ranges. This association, and that between environmental parameters and traits that affect survival^3^, suggests that endemics are rare owing to adaptive specialization and selection in favor of limited ranges of trait values and functioning (i.e. evolutionary convergence). In practice, understanding functional variability, survival mechanisms, and associated environmental contexts is necessary to inform conservation actions for such species.

Additionally, species coexist in communities, and the relative performance and competition between species is a key aspect of plant survival. Variability in functional trait values for endemic species may differ from that of sympatric (i.e. spatially co-occurring) species, which are often more widespread geographically and may exhibit greater functional diversity. Furthermore, while local environmental conditions may select for species exhibiting a convergent subset of potential trait values, finer-scale divergence within the plant community (i.e. local dissimilarity or variance in trait values) is expected as a result of adaptation to micro-scale environmental regimes (i.e. niche differentiation)^4^. Thus, we expect (1) general functional convergence within the community, but (2) functional divergence between the endemic species and non-endemic sympatric species, and (3) relatively restricted trait variances for the endemics.

Functional traits rarely operate in isolation. They usually form part of suites of characters that function together to affect survival. These suites of traits can be described as ‘ecological strategies’ or ‘adaptive strategies’, the nature of which reflects underlying trade-offs in allocation of resources available to the plant toward different functions, such as vegetative growth, regeneration, or maintenance of tissues^4^. Grime’s CSR (competitor, stress-tolerator, ruderal) plant strategy theory^4–6^ proposes that principal trade-offs faced by plants are between competitive ability (C; rapid investment in large size to allow resource preemption, which is possible only in stable, productive habitats), stress-tolerance (S; maintenance of metabolic function in variable and limiting environments), and ruderalism (R; investment in regeneration and reproduction in productive but disturbed habitats). Indeed, these dimensions are generally the main axes of trait variability in plants worldwide^7^, which can be summarized as conservative to acquisitive resource economics (equivalent to S- to R-selection), and plant size (C-selection). In practice, CSR strategies can be calculated based on the measurement of leaf traits (tissue density and leaf size measurements) that represent the end-points of these gradients of variability and the trade-offs between them^3,8,9^.

For species coexisting within plant communities, quantification and comparison of CSR strategies provide a theoretical framework for understanding functional variability that can expand interpretation beyond the variability evident for single traits^4^. Although co-occurring plant species may exhibit conspicuous convergence in CSR strategies, some studies also demonstrate fine-scale CSR strategy divergence and niche differentiation that appear to underpin coexistence^10^. Indeed, when investigated over a wide range of plant communities, CSR strategy convergence (ecological specialization) and limited species richness are evident at extremes of productivity, with high richness associated with divergence in CSR strategies at intermediate productivities^11^. Endemic species can thus be expected to exhibit CSR strategy divergence with respect to non-endemic sympatric species, but also ecological specialization evident as intraspecific convergence towards extreme strategies.

**Figure 1:**
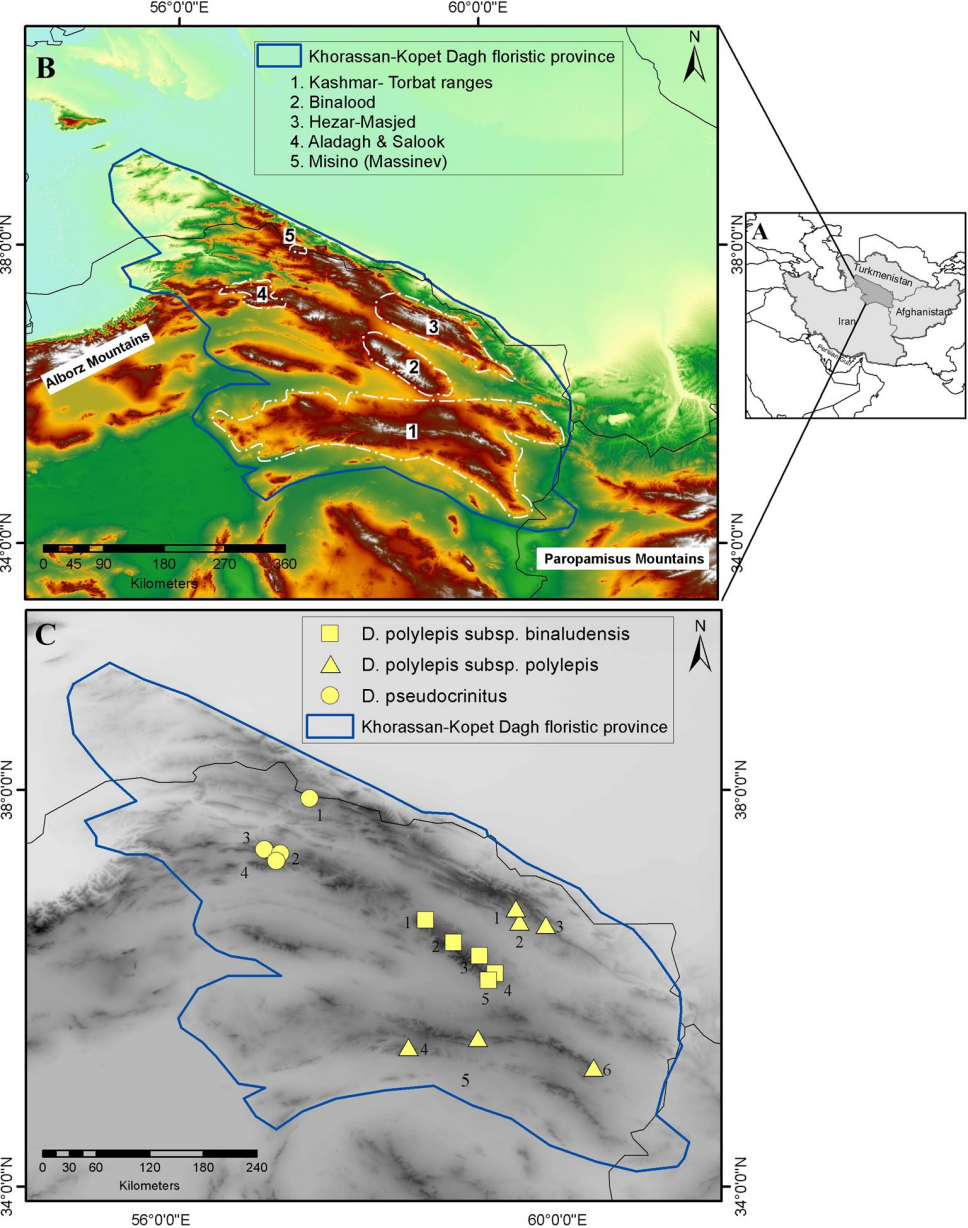
The maps of the study area and sampling sites; (**A**) geographic position of Khorassan-Kopet Dagh floristic province (KK) in northeastern Iran and southern Turkmenistan. (**B**) locations of the mountain systems in KK where samplings were carried out; (**C**) sampling sites: Stars (*Dianthus polylepis* subsp. *polylepis* sites): S1. Bezd, S2. Kardeh Dam, S3. Kuhsorkh, S4. Khowr, S5. Khomari Pass, S6. Balghur. Squares (*D. polylepis* subsp. *binaludensis* sites): S7. Zoshk, S8. Moghan, S9. Dahane Jaji, S10. Dizbad, S11. Baharkish. Circles (*Dianthus pseudocrinitus* sites): S12. Rein, S13. Misino, S14. Biu Pass, S15. Rakhtian. Prepared using ArcGIS 10.3 software (www.esri.com).

To test these hypotheses, we assessed functional compositions of communities hosting two *Dianthus* species (*Dianthus pseudocrinitus* and *Dianthus polylepis*) endemic to the montane steppes of the Khorassan-Kopet Dagh floristic province (KK) of northeastern Iran (Fig. 1; detailed in Table S1). We selected these taxa because they occur strictly in montane habitats, and can reasonably be expected to have undergone local adaption, leading to some degree of functional convergence with regard to habitat, but divergence with regard to each other. The montane steppes and associated biotas are affected and threatened by human activities, climate change, and topographic barriers^12^, and these species are thus important targets for conservation activities.

*Dianthus pseudocrinitus* Behrooz. and Joharchi is one of the endemic species in the KK floristic province restricted to a few populations in a narrow distribution. It is critically endangered (CR) according to the International Union for Conservation of Nature (IUCN) Red List categories and criteria^13^. The species is usually found in montane steppes, occurring in the calcareous mountains in northeastern Iran, at elevations of 1,600–2,300 m. *D. pseudocrinitus* nonetheless produces numerous fertile seeds and is common in disturbed habitats, both suggestive of R-selection^14^. *Dianthus polylepis* Bienert ex Boissier, though also endemic, has a broader geographic distribution, throughout the KK floristic province, and includes two nominal subspecies: *D. polylepis* subsp. *polylepis* and *D. polylepis* subsp. *binaludensis* (Rech.f.) Vaezi and Behrooz. with mostly disjunct geographic ranges. These subspecies are morphologically similar and very closely related, probably reflecting local morphological divergence^15^. *D. polylepis* subsp. *binaludensis* is restricted to the Binalood Mountains characterized by successions of sedimentary, metamorphic, and igneous rock^16^; whereas *D. polylepis* subsp. *polylepis* is distributed broadly in other Khorassan-Kopet Dagh mountains on limestone^17^. In terms of conservation status, *D. polylepis* subspecies are considered vulnerable (VU) and least concern (LC), respectively^13^. The subspecies of *D. polylepis* often occur in stressful habitats of rocky slopes, whereas *D. pseudocrinitus* is distributed patchily, in small, scattered populations with high disturbance. These ecological contrasts between closely related species allow comparisons of how different endemics function and survive that may be quite informative*.*

We focused on how functional traits and CSR strategies of these two contrasting endemic *Dianthus* species vary in range and character in their respective habitats and with respect to other, non-endemic, sympatric species. Specifically, we hypothesized that (1) endemic *Dianthus* species exhibit limited ranges of intraspecific functional variability reflecting adaptation to specific environmental conditions, and (2) the plant communities in which the endemics live exhibit functional convergence towards stress-tolerance, but interspecific functional divergence (and niche differentiation) is evident between endemic *Dianthus* species and sympatric species.

## Results

### Plant functional variability

**Figure 2:**
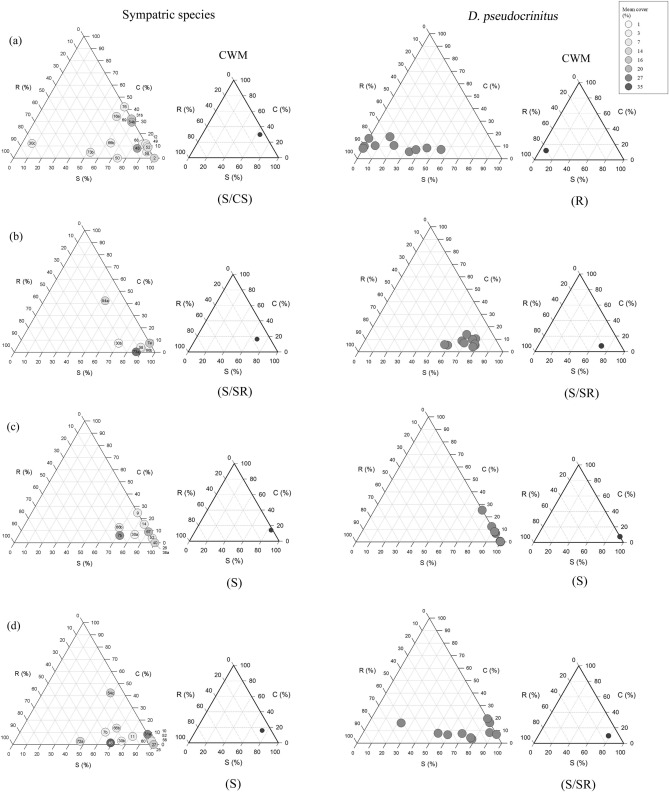
CSR classification of four sites related to *Dianthus pseudocrinitus* (**a**–**d**) showing the relative importance of the C, S and R axes for sympatric (non-*Dianthus*) species within the plant community (left side) and the individuals of *D. pseudocrinitus* (right side) in each site (**a** Rein; **b** Misino; **c** Biu Pass; **d** Rakhtian). The species are represented in gray scale according to their mean cover (%). The numbering indicated in the circles corresponds to Table S2. The small triangles show the community weighted mean (CWM) strategies at each site for the sympatric species and the individuals of *D. pseudocrinitus*.

In total, 78 species occurred (cover ≥ 5%) at the different sites, creating the set of species over which CSR strategies were assessed (Fig. 2; Table S2). A clear dominance of relatively stress-tolerant strategies was evident across the sites; indeed, most species showed a proportion of S exceeding 50% (Fig. 2, Supplementary Figs. S1, S2).

*Dianthus pseudocrinitus* was the only *Dianthus* species that exhibited general functional divergence, ranging from strong ruderalism at the Rein site (R; C:S:R = 12.0:7.2:80.8%), an intermediate strategy at Rakhtian and Misino (S/SR; C:S:R = 2.8:75.9:21.3%; and C:S:R = 7.4:70.5:22.1%, respectively), to strong stress-tolerance at the Biu Pass site (S; C:S:R = 6.8:82.3:10.9%) (Fig. 2). Differences among *D. pseudocrinitus* populations at different sites were apparent for S-selection (ANOVA on arcsine transformed data, predictor variables were sites and response variables were the percentage CSR-scores; f = 34.386, df_numerator_ = 3, df_denominator_ = 37, *p* = 0.000) and R-selection (f = 43.707, df_numerator_ = 3, df_denominator_ = 37, *p* = 0.000) but not for C-selection (f = 2.801, df_numerator_ = 3, df_denominator_ = 37, *p* = 0.054), with a Tukey’s post-hoc multiple comparison on data for R-selection (i.e. the highest f-value), suggesting that populations at all sites differed from one another, except for those at Misino and Rakhtian.

In terms of interspecific differences, analysis of variance (ANOVA) showed that *D. pseudocrinitus* differed significantly from the community mean at the Rein site in terms of R-selection (f = 46.982, df_numerator_ = 16, df_denominator_ = 146, *p* = 0.000) and S-selection (f = 44.601, df_numerator_ = 16, df_denominator_ = 146, *p* = 0.000; arcsine transformed data, with species (i.e. taxa present in the plant community) as the predictor variables and percentage CSR-scores as the response variables). Crucially, that *D. pseudocrinitus* exhibited extensive intraspecific variability was evident as extreme values of strategy variance (*s*^2^) compared to the intraspecific variability of sympatric species at the Rakhtian and Rein sites (Table 1). Note that the CSR strategy variability evident for sympatric species is presented in greater detail in Fig. S3.

**Table 1:** Variance (*s*^2^) in C-, S-, and R-selection values (%) for *D. pseudocrinitus* and other species at the (a) Rein and (b) Rakhrian sites, with species ordered according to decreasing variance in R-selection (n = 10).

Species	Variance (s^2^)		
	C-selection	S-selection	R-selection
**(a) Rein site**			
*Dianthus pseudocrinitus*	15.1	400.8	333.3
*Thymus trautvetteri*	148.4	103.2	229.4
*Tanacetum polycephalum*	101.8	150.3	195.3
*Centaurea virgata*	185.3	393.8	157.1
*Minuartia hamata*	0.0	128.1	128.1
*Stachys turcomanica*	8.0	143.8	109.3
*Melica persica*	24.4	47.3	84.4
*Bromus danthoniae*	27.0	140.3	62.9
*Lonicera iberica*	2.7	37.6	35.1
*Taeniatherum caput-medusae*	8.5	31.0	19.8
*Onobrychis cornuta*	4.5	17.5	16.3
*Cerasus pseudoprostrata*	2.7	26.9	15.8
*Phlomis cancellata*	14.4	20.4	4.3
*Acantholimon bodeanum*	0.8	1.7	0.0
*Elymus hispidus*	54.0	43.2	0.0
*Verbascum cheiranthifolium*	77.6	77.6	0.0
(**b) Rakhrian site**			
*Dianthus pseudocrinitus*	31.1	436.3	419.0
*Klasea leptoclada*	1.1	324.3	309.4
*Thymus transcaspicus*	1.2	188.2	192.8
*Artemisia kopetdaghensis*	17.0	186.6	160.4
*Stachys turcomanica*	9.7	203.6	152.5
*Dianthus orientalis* subsp. *stenocalyx*	1.3	117.9	100.6
*Boissiera squarrosa*	3.6	93.8	96.1
*Phlomis cancellata*	34.6	124.7	63.3
*Cerasus pseudoprostrata*	2.7	24.4	21.9
*Rhamnus pallasii*	1.0	1.4	2.3
*Astragalus verus*	6.5	6.5	0.0
*Crucianella gilanica*	0.0	0.0	0.0
*Elymus hispidus*	3.4	3.4	0.0
*Onobrychis cornuta*	1.7	1.7	0.0

Species authorities are reported in Table S2.

*Dianthus polylepis* subsp. *polylepis* exhibited an extreme stress-tolerant strategy (C:S:R = 0.1:99.1:0.8%) across all sites (Fig. S1). Most sympatric species at sites of *D. polylepis* subsp. *polylepis* represented a broadly stress-tolerant strategy (Fig. S1), but interspecific functional variability was evident, including subordinate species (mean cover percentage 5.5–9.0%) with relatively generalist, intermediate strategies (Fig. S1). Intraspecific differences in *Dianthus polylepis* subsp. *polylepis* between sites were apparent for C-selection (ANOVA on arcsine transformed data, predictor variables were sites and response variables the percentage CSR-scores; f = 7.599, df_numerator_ = 5, df_denominator_ = 48, *p* = 0.000) and S-selection (f = 6.686, df_numerator_ = 5, df_denominator_ = 48, *p* = 0.000) and R-selection (f = 8.099, df_numerator_ = 5, df_denominator_ = 48, *p* = 0.000), with a Tukey’s post-hoc multiple comparison on data for R-selection (i.e. the highest f-value) suggesting that the population at Bezd was distinct from other sites.

*Dianthus polylepis* subsp. *binaludensis* exhibited an extremely stress-tolerant strategy (C:S:R = 0.5:99.5:0.0%) at all sites except Zoshk, where it exhibited an intermediate S/SR strategy (Fig. S2). Intraspecific differences in *D. polylepis* subsp. *binaludensis* between sites were apparent for C-selection (ANOVA on arcsine transformed data, predictor variables were sites and response variables the percentage CSR-scores; f = 2.801, df_numerator_ = 4, df_denominator_ = 46, *p* = 0.054), S-selection (f = 25.796, df_numerator_ = 4, df_denominator_ = 46, *p* = 0.000) and R-selection (f = 18.476, df_numerator_ = 4, df_denominator_ = 46, *p* = 0.000), with a Tukey’s post-hoc multiple comparison on data for S-selection (i.e. the highest f-value) suggesting that the population at Zoshk was distinct from other sites. At Zoshk, Dahane Jaji and Dizbad, *D. polylepis* subsp. *binaludensis* exhibited significantly lower C-selection (*p* ≤ 0.05) with respect to the community mean (*t* tests within site on arcsine-transformed data).

### Site and environmental variables

**Figure 3:**
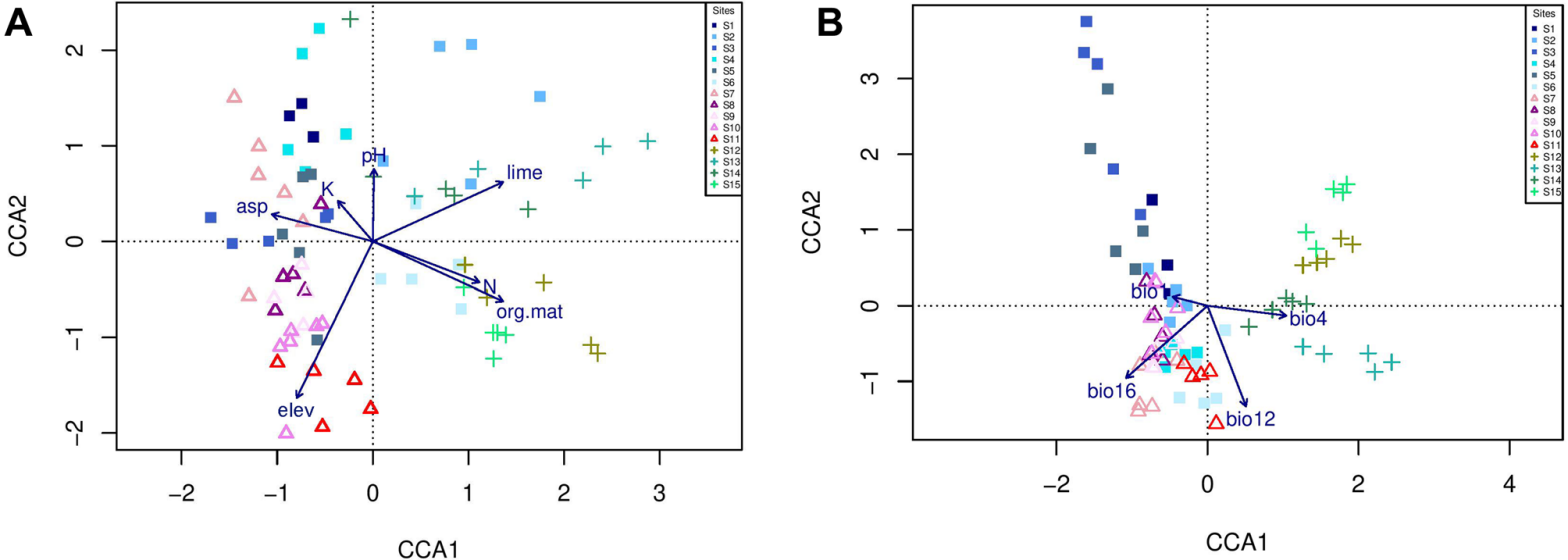
CCA ordination of the first two axes showing the distribution of the 75 plots for the 15 study sites. (**A**) soil variables (K: potassium; N: total nitrogen; org. mat: organic matter; lime: calcium carbonate) and topography (elev: elevation; asp: aspect); (**B**) bioclimatic variables (bio1: annual mean temperature; bio4: temperature seasonality; bio12: annual precipitation; bio16: precipitation of wettest quarter).

The canonical correspondence analysis (CCA) (Fig. 3) was constrained by a matrix of soil and topographic data and bioclimatic variables. Seven soil variables (clay, silt, sand, EC, P, CEC and organic carbon) and 15 bioclimatic variables were eliminated from the environmental data set owing to high collinearity (VIF > 10). Soil organic matter, pH, N, K, lime, elevation, and aspect were the edaphic/topographic variables exhibiting the highest levels of significance (*p* < 0.05; Table 2). Among the 19 bioclimatic variables, annual mean temperature (bio1), temperature seasonality (bio4), annual precipitation (bio12), and precipitation of the wettest quarter (bio16) exhibited the greatest significance. All canonical axes were significant (*p* = 0.001) (Fig. 3; Table 2).

**Table 2:** Correlations between environmental variables and the canonical correspondence analysis (CCA) ordination (see Fig. 3).

Variables	CCA1	CCA2	R^2^	Pr(> r)
**Soil factors**				
pH	0.19433	0.98094	0.1433	0.007**
N	0.79419	− 0.60767	0.361	0.001***
K	− 0.38333	0.92361	0.1411	0.004**
lime	0.99797	0.06363	0.453	0.001***
org.mat	0.77712	− 0.62935	0.5312	0.001***
elev	− 0.63087	− 0.77589	0.589	0.001***
asp	− 0.91272	− 0.40858	0.2559	0.001***
**Bioclimatic factors**				
bio1	− 0.96793	0.25122	0.0901	0.046*
bio4	0.99227	− 0.12407	0.4389	0.001***
bio12	0.35779	− 0.9338	0.8067	0.001***
bio16	− 0.74837	− 0.66328	0.8248	0.001***

Soil and topographic variables showed significant effects on species composition, with eigenvalues for the first four axes of 0.360, 0.284, 0.238, and 0.216, respectively (Fig. 3). The first four axes explained 70.1% of the total variation, with CCA1 accounting for 23.0%. The effect of elevation was greater than that of other factors (*r*^*2 *^= 0.578). For soil factors, organic matter (*r*^*2*^ = 0.514), lime (*r*^*2 *^= 0.458), and total nitrogen (*r*^*2 *^= 0.379) were the strongest explanatory variables, while pH and K showed weaker associations (Table 2). Topographic factors had greater impacts on plant species identity at sites for *D. polylepis* subsp. *binaludensis*, and elevation had the greatest *r*^2^. However, these factors were negatively associated with some sites of *D. polylepis* subsp. *polylepis* and *D. pseudocrinitus*. Thus, elevation was a major gradient in differentiating the distributions of these species. Of the environmental variables, total N, organic matter, and lime had positive correlations with all sites of *D. pseudocrinitus* and with Balghur and Kardeh Dam sites for *D. polylepis* subsp. *polylepis*. Soil nutrients, particularly total N, were the main environmental factors influencing vegetation properties at these sites.

Four bioclimatic variables had significant associations with species composition (Fig. 3B, Table 2). Precipitation of the wettest quarter (bio16) was the strongest bioclimatic variable (*r*^2 ^= 0.824), positively correlated with most sites for *D. polylepis* subsp. *binaludensis* and the Balghur and Khowre-Kalat sites for *D. polylepis* subsp. *polylepis*. Annual precipitation (bio12) and temperature seasonality (bio4; *r*^2 ^= 0.806 and 0.438, respectively) were also associated with plant community variability; both variables were positively correlated with all *D. pseudocrinitus* sites, but negatively with some sites of *D. polylepis* subsp. *polylepis* and *D. polylepis* subsp. *binaludensis*. The explanatory power of annual mean temperature (bio1) was low (*r*^2 ^= 0.090). It was positively correlated with the sites of Kardeh Dam and Bezd, for *D. polylepis* subsp. *polylepis*, and the Dizbad and Moghan sites for *D. polylepis* subsp. *binaludensis*, and negatively correlated with sites where *D. pseudocrinitus* occurred. These findings indicate considerable effects of multiple edaphic, topographic, and bioclimatic factors on the vegetation, rather than a single overarching environmental factor.

### Community CSR scores and environmental factors

These analyses revealed significant correlations between certain soil properties, bioclimatic variables, and CWM strategy scores (Table S3). We noted significant, positive correlations between the degree of C-selection (i.e. CWM-C) and several temperature and precipitation variables, as well as some negative correlations. Indeed, the strongest community-level correlations with environmental factors were between CWM-C and precipitation of driest month (bio14), precipitation seasonality (bio15), precipitation of driest quarter (bio17), and precipitation of warmest quarter (bio18), with the highest correlation coefficient (between CWM-C and temperature annual range, bio7) being 0.4428 (*r*^2 ^= 0.1150, *p* = 0.0029; Table S3). The degree of stress-tolerance in the community (CWM-S) generally showed the opposite pattern: mostly negative correlations with the same factors, although mean temperature of the wettest quarter (bio8) and precipitation of the driest month (bio14) were not significant. The extent of ruderalism in the community (CWM-R) was not correlated significantly with any climatic factors (Table S3).

Soil pH and lime content were the only soil variables that showed significant (all negative) correlations with CWM-C. In contrast, silt, pH, K, and lime showed significant positive correlations with CWM-S, and sand and P exhibited significant negative correlations (Table S3). CWM-R exhibited essentially the opposite pattern of correlations to those of CWM-S, but was positively correlated with soil N content, and not related to K and lime.

## Discussion

Our analyses revealed general functional convergence towards stress tolerance within the communities of the endemic study species, but the endemic species themselves exhibited differing degrees of functional divergence, in terms of both interspecific divergence from the community and intraspecific divergence between sites. *Dianthus pseudocrinitus* in particular showed a wide range of intraspecific functional divergence over many environmental regimes, suggesting that rarity and endemism, in this case, are not associated with intrinsic functional limitations or a lack of capacity to occupy contrasting niches—in this sense, our Hypothesis 1 was not supported. This species was found to exhibit the greatest variance (*s*^2^) in R- and S-selection of any of the co-occurring species at two sites (Table 1), and is thus relatively variable. Indeed, *D. pseudocrinitus* was found in disturbed habitats and anthropogenic sites in which soil properties had changed recently, and is capable of exhibiting strongly ruderal (R) strategies associated with higher soil total N and organic matter. The ecological strategy of *D. polylepis* is evidently different, converging on stress-tolerance (S).

If *D. pseudocrinitus* is variable and can be ruderal, why does it occupy a geographically restricted range? Why is it endemic? The genus *Dianthus* is capable of rapid radiation, with rates of 2.2–7.6 species per million years evident in Europe^18,19^ and increased diversification rates coinciding with increasing aridity during the Pleistocene^20^. *Dianthus pseudocrinitus* appears to be the closest extant relative of *D. polylepis*^14^, and thus appears to be a relatively young, “neoendemic” species. Indeed, *D. pseudocrinitus* may be extending its population size, probably via mechanisms such as polyploidy and extensive seed production (it is a tetraploid^21,22^, with 2n = 60, whereas *D. polylepis* is diploid^14^, 2n = 30).

Polyploidy is both a mechanism by which neoendemics can arise and a potential fitness advantage conferring new trait values and trait plasticities, allowing survival in heterogeneous environments^23,24^ and allowing occupation of new environments and sites. Previous studies have shown extensive unexplained phenotypic variation in some traits of *D. broteri*; high chromosome number variation in this species could be a source of phenotypic instability and variability^25^. Polyploids may have other fitness advantages over diploids: in a recent experiment, the total output of viable seed in drought and heat-stressed tetraploid plants was over four times higher than in diploids, such that tetraploids constantly produced heavier seeds with longer hygroscopic awns, traits that increase propagule fitness in extreme environments^26^.

Additionally, dispersal traits are likely a significant factor with regard to connectivity among populations in different habitats^27^. The extent to which dispersal influences a species’ geographic distribution at particular spatial scales or in specific environments depends on the mechanism of dispersal and range size^28^. *Dianthus* species exhibit short-distance seed dispersal^29,30^. *Dianthus pseudocrinitus* is unlikely to be able to disperse effectively to more distant sites and colonize suitable new regions. Indeed, species with limited dispersal occupy smaller geographic ranges because more habitable sites at the range margins remain unoccupied^28^*. Dianthus pseudocrinitus* produces heavier seeds than *D. polylepis*, due to polyploidy, suggesting that dispersal limitation is particularly important for this species. Therefore, endemic and rare species are not necessarily ecological specialists and may be restricted more by dispersal limitation and the fact that they have only recently emerged as species.

Do plant communities converge on stress-tolerance, with the endemic *Dianthus* species diverging from the rest of the plant community (Hypothesis 2)? A range of strategies was evident in the plant communities, most of which involved some degree of stress tolerance (e.g. Figure 2; Table S2), which clearly reflects the harsh environments of the study sites. Indeed, all dominant graminoids and some shrub species such as *Lonicera iberica*, *Prunus pseudoprostrata*, and *Rhamnus pallasii*, exhibited strongly stress-tolerant strategies (S and S/SR; Fig. S3). Generally, the endemic *Dianthus* species did not exhibit divergence with respect to the community mean C, S, and R values. However, at the Rein site, *D. pseudocrinitus* exhibited particularly strong variation towards R-selection and divergence from the community was statistically significant. At least, in this case, niche differentiation can be invoked as a possible mechanism for the co-existence of this species alongside other taxa. That is, our Hypothesis 2 was supported at least partially, depending on the site.

Which specific environmental factors are associated with plant adaptation in these communities? CCA indicated that both climatic (temperature and precipitation) and edaphic properties were associated with variability in CSR strategies, with greater soil N content associated particularly with ruderalism, and climatic variables particularly associated with variation between C to S selection at the community level. The plant communities at sites of *D*. *polylepis* subsp. *polylepis* and all sites of *D*. *pseudocrinitus* were influenced mainly by soil factors, whereas plant communities at sites of *D*. *polylepis* subsp. *binaludensis* were influenced more by topography. More generally, sites hosting each *Dianthus* taxon experience distinct climates (Fig. 1B). In general, factors such as low temperature, strong winds, and high solar radiation are dominant environmental stressors in mountain steppes^31^, in accordance with a general pattern of convergence towards tough, low-specific-leaf-area leaves and S-selection.

Despite the majority of the non-endemic species exhibiting convergence towards stress-tolerance, functional divergence was observed among some species adapted to disturbance. Most ruderal species occurred at Balghour and Kardeh Dam, associated with *D. polylepis* subsp. *polylepis*, and at Rein and Rakhtian for habitats of *D. pseudocrinitus*, suggestive of the operation of a disturbance at these sites. Additionally, the CCA results revealed that the distribution of vegetation among these four sites is associated with three soil factors, including N, organic matter, and lime (Fig. 3A). Indeed, although disturbance destroys live biomass, either removing it or redistributing nutrients (e.g. herbivory), or changing its form and altering nutrient cycling (e.g. fire), these effects have a significant influence on total nitrogen availability of soil and rates of net N-mineralization and net nitrification^32^. Soil changes affect plant growth and species diversity and composition, creating environments conducive to ruderals^33,34^. Paušič and Čarni^35^ pointed out that traditional land management (e.g. livestock grazing) influences the character of plant strategies, which shifted from broadly S- to broadly CR-selected. Pierce et al.^36^ also demonstrated that the relative abundance of ruderals increases with disturbance intensity, whereas the dominant stress-tolerating graminoids are suppressed. However, occasional grazing allows coexistence between potential dominants and smaller subordinates and promotes functional and species diversity by sustaining differences in the phenology of leaf growth, photosynthesis, flowering, and seed production in grasslands^37,38^. Our results suggest that increased anthropogenic disturbance is likely to have disproportionately negative impacts on populations of *D. polylepis*, which is a key consideration for the future conservation of the endemic species under study here.

These results have wider implications for the conservation of endemic species in general, because they demonstrate that being ‘endemic’ is not synonymous with being ‘incapable’ or ‘over-specialized’. Indeed, conservation of endemic species should assess whether polyploidy, neoendemism and wide variability in functional traits are evident, because species with these characteristics are likely to be capable of responding positively to habitat conservation with little extra intervention (such as supplementary ex situ conservation and population reinforcement actions). The fact that not all endemics are born equal, and that some may actually be relatively easy to conserve, is a hopeful message for plant conservationists.

## Conclusions

One endemic species, *D. pseudocrinitus*, exhibited a range of CSR strategies (R, S/SR, and S) over a range of environmental parameters, indicating that endemism, in this case, is not likely to be related to functional or ecological specialization. Rather, additional phenomena including neoendemism and dispersal limitation can be significant factors associated with rarity. Findings differed for the closely related species, *D. polylepis*. As such, each species must be evaluated on a case-by-case basis, even amongst congeneric species. These findings further suggest that endemism is not inevitably associated with vulnerability.

## Materials and methods

### Study area

Fieldwork was undertaken in different habitats across the ranges of *D. pseudocrinitus* and *D. polylepis* during three successive years (2016–2018), in the mountain steppes of northeastern Iran, between 34° 20′ and 39° 13′ N and 55° 05′ and 61° 20′ E. The area belongs to the Khorassan-Kopet Dagh floristic province (KK) in the Irano-Turanian region. The Kopet Dagh range is located in the northernmost part of the area, including the high peaks of Allaho-Akbar and Hezar-Masjed mountains. Northern ranges of Khorassan comprise the mountains of Ghorkhod, Aladagh, Salook, Shah-Jahan, and Binalood. The Sabzevar and Kashmar-Torbat ranges are oriented mainly east–west at the southern border of the KK floristic region, where Kuh-e Gar and Bezq are the highest peaks^12^ (Fig. 1A, B).

The climate is continental and the mean annual precipitation is 175–300 mm on the plains and foothills and 300–380 mm in montane regions. Precipitation falls unevenly, predominantly in late autumn, winter, and early spring (October to May), often with summer drought (June–September)^12^. The mean annual temperature varies 12–19 °C, depending on elevation^39^. The highest mean monthly air temperatures occur from June to August, with the maximum temperature rarely exceeding 45 °C. The lowest mean monthly temperatures, from December to February, can reach − 25 °C in the high mountains^40^. Most of the KK region is characterized by a Mediterranean or Irano-Turanian xeric-continental bioclimate, except for high montane areas in the central KK, where a Mediterranean or Irano-Turanian pluvi-seasonal continental bioclimate is evident, with shorter summer drought and higher annual precipitation^40,41^.

### Quadrat survey and sampling

Based on geographic distances and contrasting ecological conditions, we selected 15 sites where the endemic *Dianthus* species occur (Table S1; Fig. 1B, C). Five 5 × 5 m quadrats were established at each site, and longitude, latitude, and elevation recorded by GPS. In each quadrat, canopy cover was estimated visually as a percentage of ground area^42,43^. In total, 75 quadrats were sampled in the study area, covering much of the geographic ranges of the two endemic *Dianthus* taxa. The vascular plant species occurring within all quadrats were collected and identified, following Rechinger^44^ and Assadi et al.^45^. Accessions were deposited in the herbarium of the Ferdowsi University of Mashhad (FUMH; herbarium numbers 11011–11,386).

### Functional traits and CSR classification

We determined leaf area (LA), leaf dry-matter content (LDMC), and specific leaf area (SLA) for all species of each quadrat with a cover ≥ 5%, following the leaf area and weight measurement methodologies of Pérez-Harguindeguy et al.^46^. The material was collected from April to early July, 2016–2018, when leaves were fully expanded and mature. We selected only the most prevalent species (≥ 5% cover) in light of their greater influence on ecosystem processes^47,48^ and because performing a fully replicated functional analysis of all transitory and infrequent species with sufficient sample sizes was not feasible.

Leaf material was collected from 10 individuals of each species, packed in moist paper bags, sealed in plastic bags and stored in a thermal box until storage at 4 °C for 12–24 h. Depending on the size of leaves, 2–10 undamaged, fully expanded young leaves (including the petiole) were measured per individual (replication thus involved a mean value for each individual and 10 individual replicate plants, from which species’ means were calculated for each site). We determined the leaf area using a digital scanner and Leaf Area Measurement v1.3 software (Andrew Askew, University of Sheffield, UK). Turgid leaf fresh weight (LFW) was obtained from saturated leaves, and leaf dry weight was determined after drying for 72 h in an oven at 70 °C.

For CSR strategy analysis, values of LA, SLA, and LDMC were inserted into the ‘StrateFy’ spreadsheet^3^ to calculate C, S, and R percentages for each species. CSR coordinates were plotted in ternary plots using SigmaPlot version 10 (Systat Software Inc., San Jose, California, USA). Community-weighted mean (CWM) values for C, S, and R scores were calculated for each site using species’ mean CSR score values weighted by their relative cover^10,49^, using R 3.5.0 with the *FD* package^49,50^. Correlations between CWM-CSR and environmental variables were tested using Pearson correlation coefficients. Simple linear regressions of each of the three scores on each of the 33 environmental variables were calculated to test significance, in R.

### Environmental factors

Environmental data encompassing soil type, topography, and climate were recorded for each quadrat. Soil samples (~ 500 g) were taken at a depth of 15–25 cm, placed in a polyethylene bag, labeled, and transported to the laboratory. The following properties were measured: pH, electrical conductivity (EC), cation-exchange capacity (CEC), organic carbon, organic matter, total nitrogen, K, P, lime, silt, sand, and clay percentage.

Bioclimatic variables were extracted from the WorldClim global climate database, with a 30″ spatial resolution^51^. The latitude and longitude of each quadrat were recorded in the software ArcGIS 10.3.1., and corresponding values of the bioclimatic variables were extracted for each quadrat site (Table S4).

### Statistical analysis

Canonical correspondence analysis (CCA)^52^ was used to explore relationships between sites and environmental factors. Variance inflation factors (VIF) of variables were used to quantify how much a regression coefficient is inflated by the presence of other explanatory variables. Collinear environmental variables with high variance inflation factors (> 10) were eliminated from further analyses. Finally, we used a permutation test (999 permutations, *p* < 0.05) to test significance of the *R*^2^ for each environmental variable with respect to the canonical axes of CCA. All of these analyses were conducted using R 3.5.0 software^53^ with the *vegan* package^54^.

## Supplementary information

Supplementary Information.

## Data Availability

Data used in conducting this study are available for researchers upon request to the corresponding author for reasonable use in research.
