# Supplementary material for: Are endemic species necessarily ecological specialists? Functional variability and niche differentiation of two threatened Dianthus species in the montane steppes of northeastern Iran
Source: Sci Rep. 2020 Jul 16;10:11774. doi: 10.1038/s41598-020-68618-7 (PMC7366929; doi:10.1038/s41598-020-68618-7)
Supplement: Supplementary file 1 — Supplementary Information. [file 41598_2020_68618_MOESM1_ESM.docx]

**Title Page**

**Are endemic species necessarily ecological specialists?**

**Functional variability and niche differentiation of two threatened *Dianthus* species in the montane steppes of northeastern Iran**

**Maryam Behroozian^1^, Hamid Ejtehadi^1*^, Farshid Memariani^2^, Simon Pierce^3^, Mansour Mesdaghi^4^**

**Supporting Information**

**Are endemic species necessarily ecological specialists?**

**Functional variability and niche differentiation of two threatened *Dianthus* species in the montane steppes of northeastern Iran**

**Supporting Information captions**

**Fig. S1.** CSR classification of six sites related to *Dianthus polylepis* subsp. *polylepis* (a-f) showing the relative importance of the C, S and R axes for sympatric (non-*Dianthus*) species within the plant community (left side) and the individuals of *D. polylepis* subsp. *polylepis* (right side) in each site (a. Bezd; b. Kardeh Dam; c. Kuhsorkh; d. Khowre- Kalat; e. Khomari Pass; f. Balghour). The species are represented in gray scale according to their mean cover (%). The numbering indicated in the circles corresponds to Table 2. The small triangles show the community weighted mean (CWM) strategies at each site for the sympatric species and the individuals of *D. polylepis* subsp. *polylepis.*

**Fig. S2.** CSR classification of five sites related to *Dianthus polylepis* subsp. *binaludensis* (a-e) showing the relative importance of the C, S and R axes for sympatric (non-*Dianthus*) species within the plant community (left side) and the individuals of *D. polylepis* subsp. *binaludensis* (right side) in each site (a. Zoshk; b. Moghan; c. Dahane Jaji; d. Dizbad; e. Baharkish). The species are represented in gray scale according to their mean cover (%). The numbering indicated in the circles corresponds to Table 2. The small triangles show the community weighted mean (CWM) strategies at each site for the sympatric species and the individuals of *D. polylepis* subsp. *binaludensis.*

**Fig. S3.** Intraspecific CSR variability of the most dominant species at the sites of the three *Dianthus* taxa. Individuals from different locations (i.e. different sites of each endemic *Dianthus* species) are represented in different shades of gray. Continued over -

**Fig. S3.** Continued: *Stachys turcomanica* was the only dominant endemic species in the Irano-Turanian region, occurring in all sites for *D. pseudocrinitus*; it also displayed high intraspecific variability among its individuals, from S to S/SR across sites. The presence of ruderals such as *D. pseudocrinitus* (R) and *Thymus trautvetteri* (SR) at Rein confirms the relatively disturbed environment. Although the most dominant species at these sites were stress-tolerators, some dominant species (e.g. *Thymus transcaspicus*, *Stachys turcomanica*, *Minuartia hamata*) exhibited intermediate strategies (SR, S/SR) across sites; as such, the results reflected a consistent shift towards disturbance. At sites with *D. pseudocrinitus*, some species exhibited different strategies across sites, e.g. *Phlomis cancellata* (SC/CSR, S/SC, SC), *Thymus trautvetteri* (S, SR), *Stachys turcomanica* (S, S/SR) and *Artemisia kopetdaghensis* (S, S/SR). *Klasea leptoclada* is a regional endemic and the most dominant species (mean cover = 35%) among all dominant species at sites of *D. pseudocrinitus*. It exhibited a stress-tolerant strategy (S/SR; C:S:R = 1.3 : 68.9 : 29.8%); in terms of intraspecific variability, individuals of this species varied from S to S/SR, such that *K. leptoclada* exhibited less variability than *D. pseudocrinitus*. The second most dominant species (25% cover) was *Thymus trautvetteri*, which exhibited an extreme stress-tolerant strategy at the Misino site (S; C:S:R = 0.3 : 86.5 : 13.2%) and an intermediate stress-tolerant strategy with less mean cover percentage (10%) at the Rein site (SR; C:S:R = 4.2 : 76.6 : 19.0%). The third most dominant species (*Elymus hispidus*; 19.5%) displayed an extremely stress-tolerant strategy (C:S:R = 8.3 : 91.7 : 0.0%) at the Rakhtian site, and the fourth most dominant species (*Lonicera iberica*, 15.8%) exhibited a highly stress-tolerant strategy (C:S:R = 7.9 : 84.0 : 8.1%) at the Rein site. Stress-tolerant competitors (SC) included only two species: *Phlomis cancellata* (C:S:R = 42.3 : 48.2 : 9.5%) and *Verbascum cheiranthifolium* (C:S:R = 42.0 : 58.0 : 0.0%). Dominant species at sites of *D. polylepis* subsp. *polylepis*, such as *Acantholimon spinicalyx* and *Artemisia kopetdaghensis*, with the greatest mean cover (20% and 18%, respectively), exhibited highly stress-tolerant strategies (respectively, C:S:R = 0.5 : 95.5 : 0.0% and C:S:R = 2.6 : 93.2 : 4.2%), respectively. Other prevalent species, (mean cover ~ 15%, included *Astragalus verus* (C:S:R = 10.3 : 89.7 : 0.0%), *Rosa persica* (C:S:R = 2.4 : 97.6 : 0.0%), and *Klasea latifolia* (C:S:R = 42.3 : 57.2 : 0.5%). All of these species are also endemic to the Irano-Turanian (IT) region and the KK floristic province which displayed more or equal intraspecific variability than *D. polylepis* subsp. *polylepis*. *Lactuca orientalis* was the only species that showed an intermediate strategy between C and R, and for one site only (Khomari). *Euphorbia microsciadia* exhibited an intermediate strategy between S and R (C:S:R = 9.2 : 55.7 : 35.1 %), and intermediate strategies between S and C were also apparent, such as in the stress-tolerant competitors *Cousinia chaetocephala* (C:S:R = 42.0 : 57.2 : 0.8%) at Balghour and Kardeh; *Cousinia freynii* (C:S:R = 42.6 : 57.4 : 0.0%) at Balghour, Kuhsorkh and Khowre-Kalat; and *Klasea latifolia* (C:S:R = 42.3 : 57.2 : 0.5%) in Kuhsorkh and Kardeh Dam. The other two intermediate strategies were exhibited by *Onobrychis verae* and *Poa bulbosa* as S/SC (C:S:R = 19.0 : 81.0 : 0.0%, with 14% cover) at Kardeh Dam and S/SR (C:S:R=0.2 : 78.6 : 21.2%, 14% cover) at Kardeh Dam and Khowre-Kalat, respectively. Two relatively competitive species were evident: *Cousinia eryngioides* (C:S:R=62.5 : 37.5 : 0.0%, 4% cover) at Kardeh Dam and *Eryngium bungei* (C:S:R=70.1 : 8.5 : 21.4%, 5.5% cover) at Kuhsorkh. Most species at sites hosting *D. polylepis* subsp. *binaludensis* exhibited a general interspecific functional divergence, ranging from extreme competitors (C) through stress-tolerant competitors (SC) to broadly stress-tolerant (S) species. Highly competitive species included *Cousinia discolor* (C:S:R = 85.4 : 1.3 : 13.3%, 15% cover) at Zoshk, and *Ferula ovina* (C:S:R = 87.9 : 12.1 : 0.0%, 10.7%) at Dahane Jaji. Furthermore, ruderal species evident at Zoshk included *Hymenocrater* *elegans* (C:S:R = 30.4 : 0.0 : 69.6%, 25%) and *Silene swertiifolia* (C:S:R = 31.3 : 0.0 : 68.7%, 6%). The dominant species at these different sites exhibited various strategies: e.g., *Centaurea virgata* was S, SR/CSR, and *Cousinia discolor* was S/CS, CS. *Festuca valesiaca* was the most dominant species, with a widespread distribution among stress-tolerator species in Baharkish (mean cover 30%), which exhibited extreme stress-tolerance (C:S:R = 6.2 : 93.8 : 0.0%). The second most dominant species (*Varthemia persica*; 25% cover) exhibited an intermediate S/SR strategy at the Zoshk site, where the community was relatively ruderal. It was the only dominant species that exhibited high intraspecific variability, from S/SR to SR. Dizbad was the second site where the community exhibited a high degree of intermediate strategies.

**Table S1.** Location details of the 15 sites in the study area.

**Table S2**. List of the dominant plant species and their tertiary CSR categories, collected from the 15 study sites associated with *Dianthus* species. Repetition of species with different letters (a, b, c, d) represents measurements from more than one site.

**Table S3**. Relationships between CWM-CSR and environmental variables from a Pearson’s correlation coefficient and significance test using linear regressions.

**Table S4.** Environmental variables used in the study. Climate data were obtained from WorldClim ([www.WorldClim.org](http://www.WorldClim.org)).


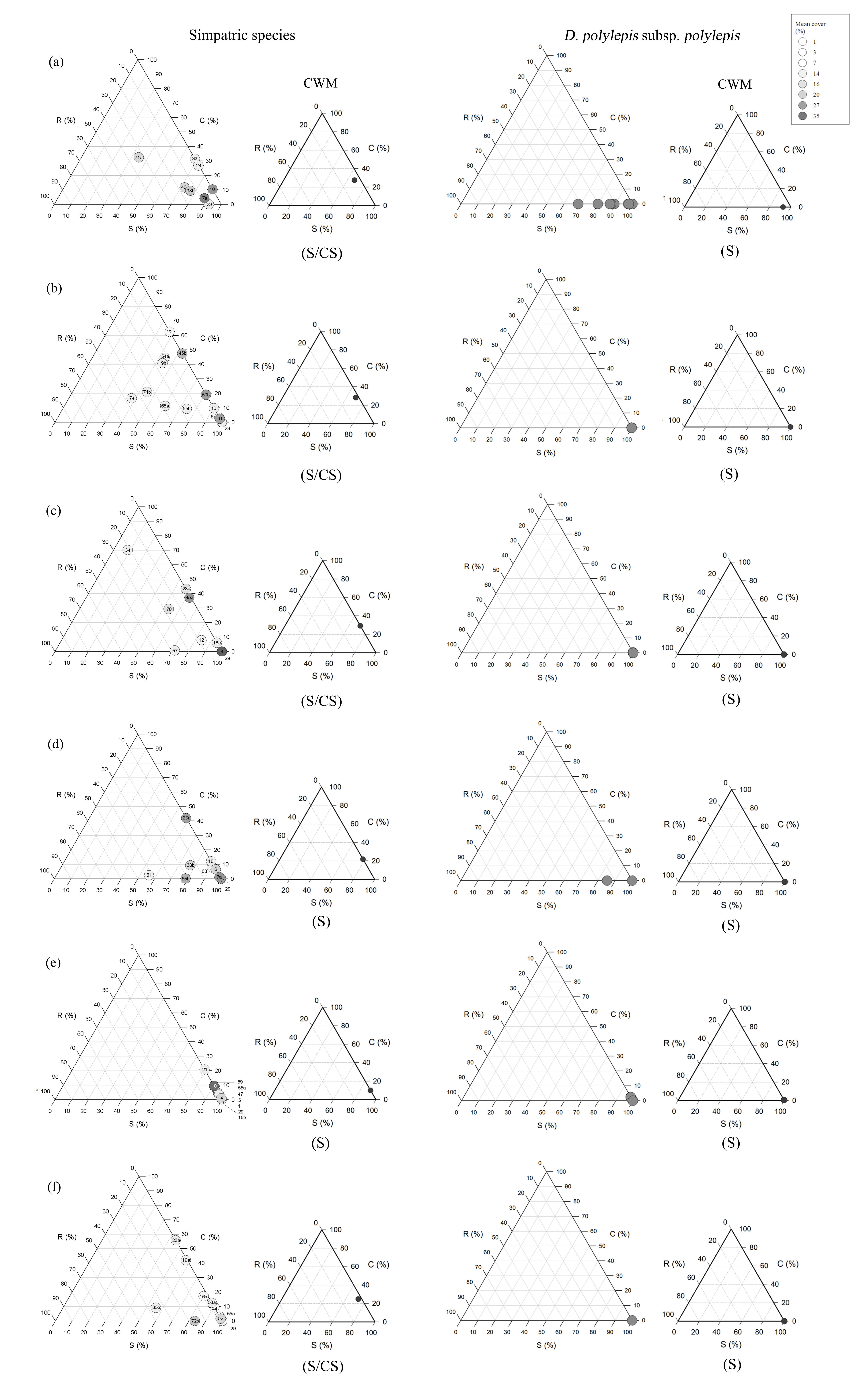


**Fig. S1.** CSR classification of six sites related to *Dianthus polylepis* subsp. *polylepis* (a-f) showing the relative importance of the C, S and R axes for sympatric (non-*Dianthus*) species within the plant community (left side) and the individuals of *D. p.* subsp. *polylepis* (right side) in each site (a. Bezd; b. Kardeh Dam; c. Kuhsorkh; d. Khowre- Kalat; e. Khomari Pass; f. Balghour). The species are represented in gray scale according to their mean cover (%). The numbering indicated in the circles corresponds to Table S2. The small triangles show the community weighted mean (CWM) strategies at each site for the sympatric species and the individuals of *D. p.* subsp. *polylepis.*


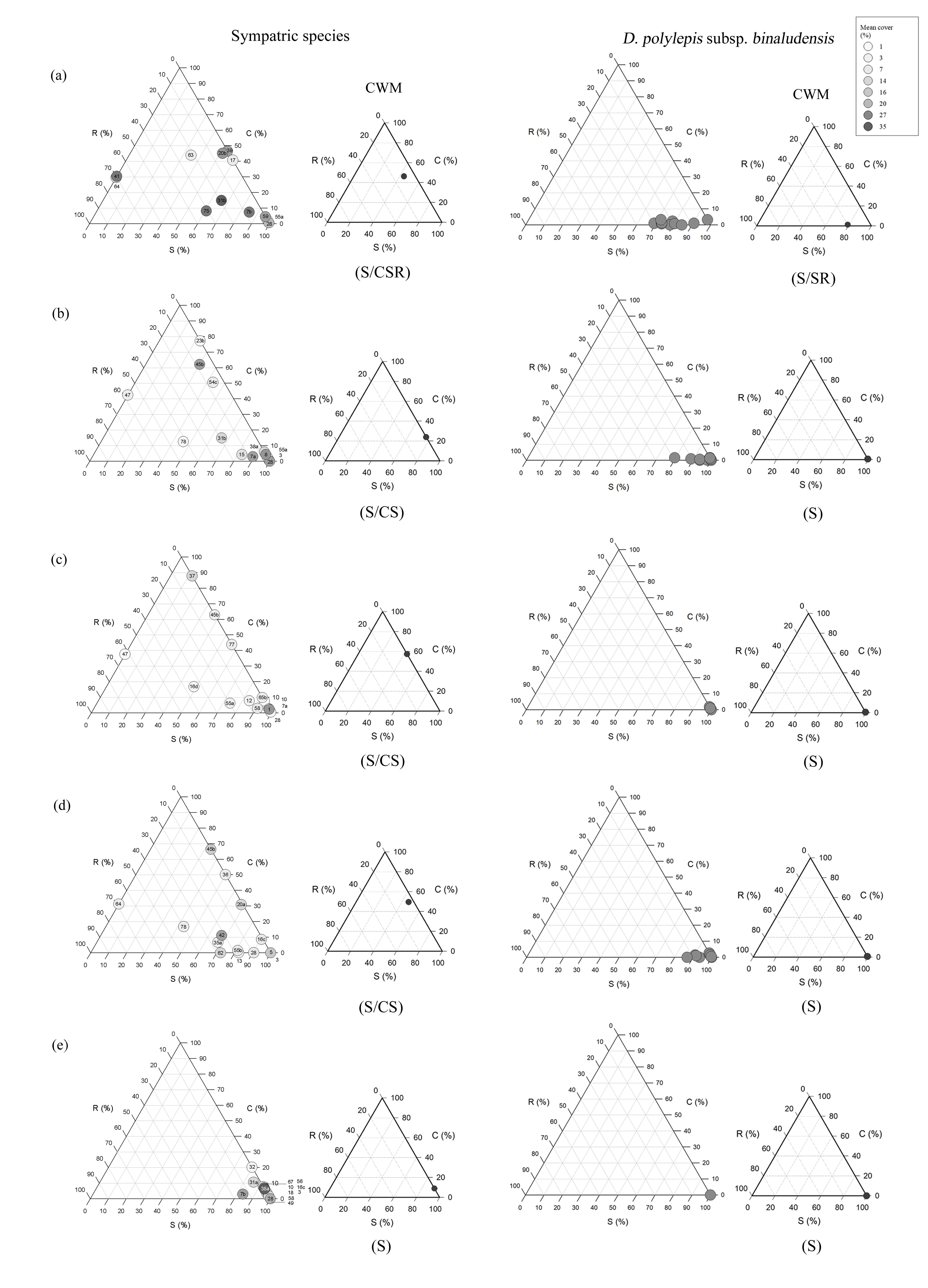


**Fig. S2.** CSR classification of five sites related to *Dianthus polylepis* subsp. *binaludensis* (a-e) showing the relative importance of the C, S and R axes for sympatric (non-*Dianthus*) species within the plant community (left side) and the individuals of *D. polylepis* subsp. *binaludensis* (right side) in each site (a. Zoshk; b. Moghan; c. Dahane Jaji; d. Dizbad; e. Baharkish). The species are represented in gray scale according to their mean cover (%). The numbering indicated in the circles corresponds to Table S2. The small triangles show the community weighted mean (CWM) strategies at each site for the sympatric species and the individuals of *D. polylepis* subsp. *binaludensis.*





**Fig. S3.** Intraspecific CSR variability of the most dominant species at the sites of the three *Dianthus* taxa. Individuals from different locations (i.e. different sites of each endemic *Dianthus* species) are represented in different shades of gray. Continued over -

**Fig. S3.** Continued: *Stachys turcomanica* was the only dominant endemic species in the Irano-Turanian region, occurring in all sites for *D. pseudocrinitus*; it also displayed high intraspecific variability among its individuals, from S to S/SR across sites. The presence of ruderals such as *D. pseudocrinitus* (R) and *Thymus trautvetteri* (SR) at Rein confirms the relatively disturbed environment. Although the most dominant species at these sites were stress-tolerators, some dominant species (e.g. *Thymus transcaspicus*, *Stachys turcomanica*, *Minuartia hamata*) exhibited intermediate strategies (SR, S/SR) across sites; as such, the results reflected a consistent shift towards disturbance. At sites with *D. pseudocrinitus*, some species exhibited different strategies across sites, e.g. *Phlomis cancellata* (SC/CSR, S/SC, SC), *Thymus trautvetteri* (S, SR), *Stachys turcomanica* (S, S/SR) and *Artemisia kopetdaghensis* (S, S/SR). *Klasea leptoclada* is a regional endemic and the most dominant species (mean cover = 35%) among all dominant species at sites of *D. pseudocrinitus*. It exhibited a stress-tolerant strategy (S/SR; C:S:R = 1.3 : 68.9 : 29.8%); in terms of intraspecific variability, individuals of this species varied from S to S/SR, such that *K. leptoclada* exhibited less variability than *D. pseudocrinitus*. The second most dominant species (25% cover) was *Thymus trautvetteri*, which exhibited an extreme stress-tolerant strategy at the Misino site (S; C:S:R = 0.3 : 86.5 : 13.2%) and an intermediate stress-tolerant strategy with less mean cover percentage (10%) at the Rein site (SR; C:S:R = 4.2 : 76.6 : 19.0%). The third most dominant species (*Elymus hispidus*; 19.5%) displayed an extremely stress-tolerant strategy (C:S:R = 8.3 : 91.7 : 0.0%) at the Rakhtian site, and the fourth most dominant species (*Lonicera iberica*, 15.8%) exhibited a highly stress-tolerant strategy (C:S:R = 7.9 : 84.0 : 8.1%) at the Rein site. Stress-tolerant competitors (SC) included only two species: *Phlomis cancellata* (C:S:R = 42.3 : 48.2 : 9.5%) and *Verbascum cheiranthifolium* (C:S:R = 42.0 : 58.0 : 0.0%). Dominant species at sites of *D. polylepis* subsp. *polylepis*, such as *Acantholimon spinicalyx* and *Artemisia kopetdaghensis*, with the greatest mean cover (20% and 18%, respectively), exhibited highly stress-tolerant strategies (C:S:R = 0.5 : 95.5 : 0.0% and C:S:R = 2.6 : 93.2 : 4.2%), respectively. Other prevalent species, (mean cover ~ 15%, included *Astragalus verus* (C:S:R = 10.3 : 89.7 : 0.0%), *Rosa persica* (C:S:R = 2.4 : 97.6 : 0.0%), and *Klasea latifolia* (C:S:R = 42.3 : 57.2 : 0.5%). All of these species are also endemic to the Irano-Turanian (IT) region which displayed more or equal intraspecific variability than *D. polylepis* subsp. *polylepis*. *Lactuca orientalis* was the only species that showed an intermediate strategy between C and R, and for one site only (Khomari). *Euphorbia microsciadia* exhibited an intermediate strategy between S and R (C:S:R = 9.2 : 55.7 : 35.1 %), and intermediate strategies between S and C were also apparent, such as in the stress-tolerant competitors *Cousinia chaetocephala* (C:S:R = 42.0 : 57.2 : 0.8%) at Balghour and Kardeh; *Cousinia freynii* (C:S:R = 42.6 : 57.4 : 0.0%) at Balghour, Kuhsorkh and Khowre-Kalat; and *Klasea latifolia* (C:S:R = 42.3 : 57.2 : 0.5%) in Kuhsorkh and Kardeh Dam. The other two intermediate strategies were exhibited by *Onobrychis verae* and *Poa bulbosa* as S/SC (C:S:R = 19.0 : 81.0 : 0.0%, with 14% cover) at Kardeh Dam and S/SR (C:S:R=0.2 : 78.6 : 21.2%, 14% cover) at Kardeh Dam and Khowre-Kalat, respectively. Two relatively competitive species were evident: *Cousinia eryngioides* (C:S:R=62.5 : 37.5 : 0.0%, 4% cover) at Kardeh Dam and *Eryngium bungei* (C:S:R=70.1 : 8.5 : 21.4%, 5.5% cover) at Kuhsorkh. Most species at sites hosting *D. polylepis* subsp. *binaludensis* exhibited a general interspecific functional divergence, ranging from extreme competitors (C) through stress-tolerant competitors (SC) to broadly stress-tolerant (S) species. Highly competitive species included *Cousinia discolor* (C:S:R = 85.4 : 1.3 : 13.3%, 15% cover) at Zoshk, and *Ferula ovina* (C:S:R = 87.9 : 12.1 : 0.0%, 10.7%) at Dahane Jaji. Furthermore, ruderal species evident at Zoshk included *Hymenocrater* *elegans* (C:S:R = 30.4 : 0.0 : 69.6%, 25%) and *Silene swertiifolia* (C:S:R = 31.3 : 0.0 : 68.7%, 6%). The dominant species at these different sites exhibited various strategies: e.g., *Centaurea virgata* was S, SR/CSR, and *Cousinia discolor* was S/CS, CS. *Festuca valesiaca* was the most dominant species, with a widespread distribution among stress-tolerator species in Baharkish (mean cover 30%), which exhibited extreme stress-tolerance (C:S:R = 6.2 : 93.8 : 0.0%). The second most dominant species (*Varthemia persica*; 25% cover) exhibited an intermediate S/SR strategy at the Zoshk site, where the community was relatively ruderal. It was the only dominant species that exhibited high intraspecific variability, from S/SR to SR. Dizbad was the second site where the community exhibited a high degree of intermediate strategies.

**Table S1.** Location details of the 15 sites in the study area for three endemic *Dianthus* taxa.

| Species | Site name | Site name | Elevation range (m) | Latitude | Longitude |
| --- | --- | --- | --- | --- | --- |
| *D. polylepis* subsp. *polylepis* | S1 | Bezd | 1505-1525 | 35° 11′ 48.1″ | 60° 21′ 33.8″ |
|  | S2 | Kardeh Dam | 1475-1491 | 36° 40′ 19.9″ | 59° 36′ 43.9″ |
|  | S3 | Kuhsorkh | 1493-1520 | 35° 24′ 19.1″ | 58° 29′ 25.1″ |
|  | S4 | Khowre- Kalat | 1776-1799 | 36° 38′ 20.0″ | 59° 52′ 45.1″ |
|  | S5 | Khomari Pass | 1855-1893 | 35° 29′ 47.4″ | 59° 11′ 36.2″ |
|  | S6 | Balghour | 1794-1803 | 36° 48′ 18.7″ | 59° 34′ 29.6″ |
| *D. polylepis* subsp. *binaludensis* | S7 | Zoshk | 1793-1822 | 36° 19′ 54.1″ | 59° 12′ 38.2″ |
|  | S8 | Moghan | 1964-1974 | 36° 09′ 11.2″ | 59° 22′ 06.6″ |
|  | S9 | Dahane Jaji | 1796-1970 | 36° 28′ 45.8″ | 58° 57′ 22.0″ |
|  | S10 | Dizbad | 2007-2048 | 36° 05′ 01.0″ | 59° 18′ 00.7″ |
|  | S11 | Baharkish | 2179-2245 | 36° 41′ 31.2″ | 58° 40′ 03.7″ |
| *D. pseudocrinitus* | S12 | Rein | 1871-1899 | 37° 24′ 06.8″ | 57° 02′ 27.6″ |
|  | S13 | Misino | 1647-1695 | 37° 54′ 53.3″ | 57° 29′ 44.9″ |
|  | S14 | Biu Pass | 1664-1674 | 37° 21′ 31.7″ | 57° 11′ 56.4″ |
|  | S15 | Rakhtian | 1880-1923 | 37° 17′ 11.0″ | 57° 08′ 59.3″ |

**Table S2**. List of the dominant plant species and their tertiary CSR categories, collected from the 15 study sites associated with *Dianthus* species. Repetition of species with different letters (a, b, c, d) represents measurements from more than one site.

| No. | Species name | CSR | Chorotype | No. | Species name | CSR | Chorotype |
| --- | --- | --- | --- | --- | --- | --- | --- |
| 1 | *Acantholimon avenaceum* Bunge | S | KK ^Omni *^ | 40 | *Hedysarum longipedunculatum* Ranjbar & Karamian | SC | IT ^KK-Alborz **^ |
| 2 | *Acantholimon bodeanum* Bunge | S | IT ^KK - Alborz **^ | 41 | *Hymenocrater* *elegans* Bunge | R/CR | IT ^KK-Alborz *^ |
| 3 | *Acantholimon erinaceum* (Jaub. & Spach) Lincz. | S | IT ^C&E^ | 42 | *Hymenocrater oxyodontus* Rech.f. | S/SR | IT ^C *^ |
| 4 | *Acantholimon spinicalyx* Koeie & Rech.f. | S | KK ^E-S^ ^**^ | 43 | *Hymenocrater platystegius* Rech.f. | S/SR | IT ^KK **^ |
| 5 | *Acanthophyllum glandulosum* Buhse ex Boiss. | S | IT ^C&E^ | 44 | *Jurinea sintenesii* Bornm. | S | IT ^KK *^ |
| 6 | *Agropyron cristatum* (L.) Gaertn. | S | PL | 45a | *Klasea latifolia* (Boiss.) L.Martins | CS | IT ^C^ |
| 7a | *Artemisia kopetdaghensis* Krasch. Popov & Lincz. ex Poljakov | S | KK ^Omni^ ^(exc. NW)*^ | 45b | *Klasae latifolia* (Boiss.) L.Martins | C/CS | IT ^C^ |
| 7b | *Artemisia kopetdaghensis* Krasch. Popov & Lincz. ex Poljakov | S/SR | KK ^Omni^ ^(exc. NW)*^ | 46 | *Klasea leptoclada* (Bornm. & Sint.) L.Martins | S/SR | KK ^W-C^ * |
| 8 | *Astragalus floccosus* Boiss. | S |  | 47 | *Lactuca orientalis* (Boiss.) Boiss. | CR | IT |
| 9 | *Astragalus pendulinus* Popov. & B.Fedtsch | S/SC | KK ^Omni^ ^(exc. S)^ ^*^ | 48 | *Lonicera iberica* M.Bieb. | S | IT ^Cauc -Turk.^ |
| 10 | *Astragalus verus* Olivier | S | IT ^C^ | 49 | *Melica persica* Kunth | S | IT |
| 11 | *Boissiera squarrosa* (Banks & Sol.) Nevski | S | IT-M | 50 | *Minuartia hamate* (Hausskn.) Mattf. | S/SR | IT-M |
| 12 | *Bromus danthoniae* Trin. | S | PL | 51 | *Noccaea trinervia* Steud. | SR | IT |
| 13 | *Bromus tectrom* L. | S/SR | PL | 52 | *Onobrychis cornuta* (L.) Desv. | S | IT ^KK-E^ |
| 14 | *Bupleurum falcatum* L. subsp. *cernuum* (Ten.) Arcang. | S | IT ^Omni^ | 53a | *Onobrychis verae* Sirj. | S | IT ^KK-Afgh. *^ |
| 15 | *Carex stenophylla* Wahlenb. | S/SR | PL | 53b | *Onobrychis verae* Sirj. | S/CS | IT ^KK-Afgh. *^ |
| 16a | *Centaurea virgata* subsp. *squarrosa* (Willd.) Gugler | SC | IT | 54a | *Phlomis cancellata* Bunge | SC/CSR | IT ^KK-Afgh. *^ |
| 16b | *Centaurea virgata* subsp. *squarrosa* (Willd.) Gugler | S/SC | IT | 54b | *Phlomis cancellata* Bunge | S/CS | IT ^KK-Afgh. *^ |
| 16c | *Centaurea virgata* subsp. *squarrosa* (Willd.) Gugler | S | IT | 54c | *Phlomis cancellata* Bunge | SC | IT ^KK-Afgh. *^ |
| 16d | *Centaurea virgata* subsp. *squarrosa* (Willd.) Gugler | SR/CSR | IT | 55a | *Poa bulbosa* L. | S | IT-ES-M |
| 17 | *Cirsium congestum* Fisch. & C.A. Mey. ex DC. | SC |  | 55b | *Poa bulbosa* L. | S/SR | IT-ES-M |
| 18 | *Cotoneaster nummularius* Fisch. & C.A.Mey. | S | IT | 56 | *Poa versicolor Besser* subsp. *araratica* (Trautv.) Tzvelev | S | IT |
| 19a | *Cousinia chaetocephala* Kult. | CS | KK ^C-E **^ | 57 | *Polygonum paronychioides* C.A.Mey. | S/SR | IT |
| 19b | *Cousinia chaetocephala* Kult. | CS/CSR | KK ^C-E **^ | 58 | *Prunus pseudoprostrata* (Pojark.) Rech.f. | S | IT ^KK-Alborz^ |
| 20a | *Cousinia discolor* Bunge | S/CS | KK ^E *^ | 59 | *Prunus turcomanica* (Pojark.) Gilli | S | KK ^Omni *^ |
| 20b | *Cousinia discolor* Bunge | CS | KK ^E^ * | 60 | *Rhamnus pallasii* Fisch. & C.A.Mey. | S | IT-ES |
| 21 | *Cousinia elata* Boiss. & Buhse | S/CS | IT ^KK-Alborz *^ | 61 | *Rosa persica* Michx. ex Juss. | S | IT ^C & E^ |
| 22 | *Cousinia eryngioides* Boiss. | C/CS | IT ^C *^ | 62 | *Salvia abrotanoides* (Kar.) Sytsma | S/CS | IT ^C & E^ |
| 23a | *Cousinia freynii* Bornm. | CS | KK ^W-C-E*^ | 63 | *Sanguisorba* *minor* Scop. | CS/CSR | IT-ES-M |
| 23b | *Cousinia freynii* Bornm. | C/CS | KK ^W-C-E^* | 64 | *Silene swertiifolia* Boiss. | R/CR | IT |
| 24 | *Cousinia platyraphis* Kult. | S/CS | KK ^C-S **^ | 65a | *Stachys lanvadulifolia* Vahl | S/CSR | IT ^C^ |
| 25 | *Crucianella gilanica* subsp. *transcaspica* (Ehrend.) Ehrend. & Schönb. -Tem | S | IT | 65b | *Stachys lavandulifolia* Vahl | S | IT ^C^ |
| 26 | *Crucianella sintenisii* Bornm. | S | KK ^NW-W-C^ ^**^ | 66a | *Stachys turcomanica* Trautv. | S | IT ^KK – Alborz *^ |
| 27 | *Dianthus orientalis* subsp. *stenocalyx* (Boiss.) Rech.f. | S | IT ^C **^ | 66b | *Stachys turcomanica* Trautv. | S/SR | IT ^KK – Alborz *^ |
| 28 | *Dianthus polylepis* subsp. *binaludensis* (Rech.f.) Vaezi & Behrooz. | S | KK ^C-E^** | 67 | *Stipa arabica* Trin. & Rupr. | S | IT |
| 29 | *Dianthus polylepis* subsp. *polylepis* | S | KK ^C-E-S *^ | 68 | *Taeniatherum caput-medusae* (L.) Nevski | S | IT-ES-M |
| 30a | *Dianthus pseudocrinitus* Behrooz. & Joharchi | S | KK ^W^** | 69 | *Tanacetum polycephalum* Sch. –Bip. | S/CS | IT ^W & C^ |
| 30b | *Dianthus pseudocrinitus* Behrooz. & Joharchi | S/SR | KK ^W^ ** | 70 | *Thalictrum isopyroides* C.A.Mey. | S/CSR | IT ^Omni^ |
| 30c | *Dianthus pseudocrinitus* Behrooz. & Joharchi | R | KK ^W **^ | 71a | *Thalictrum sultanabadense* Stapf | CSR | IT ^C^ |
| 31a | *Elymus hispidus* (Opiz) Melderis | S | IT-ES-M | 71b | *Thalictrum sultanabadense* Stapf | SR/CSR | IT ^C^ |
| 31b | *Elymus hispidus* (Opiz) Melderis | S/SR | IT-ES-M | 72a | *Thymus transcaspicus* Klokov. | SR | KK ^W-C-E^ * |
| 32 | *Eremurus spectabilis* M.Bieb. | S/CS | IT | 72b | *Thymus transcaspicus* Klokov. | S/SR | KK ^W-C-E^* |
| 33 | *Eryngium billardieri* F.Delaroche | S/CS | IT ^C^ | 73a | *Thymus trautvetteri* Klokov. & Desj. –Shost. | S | IT ^Cauc. - Turk.^ |
| 34 | *Eryngium bungei* Boiss. | C/CR | IT ^C^ | 73b | *Thymus trautvetteri* Klokov. & Desj. –Shost. | SR | IT ^Cauc. - Turk.^ |
| 35a | *Euphorbia microsciadia* Boiss. | S/SR | IT ^C^ | 74 | *Trigonella subenervis* Rech.f. | SR/CSR | KK ^C-E-S **^ |
| 35b | *Euphorbia microsciadia* Boiss. | SR | IT ^C^ | 75 | *Varthemia persica* D.C. | S/SR | IT ^C^ |
| 36 | *Ferula flobelliloba* Rech.f. & Aellen | CS | KK ^C-E **^ | 76 | *Verbascum cheiranthifolium* Boiss. | CS | IT ^W & C^ |
| 37 | *Ferula ovina* (Boiss.) Boiss. | C | IT ^C & E^ | 77 | *Verbascum speciosum* Schrad. | CS | IT- M |
| 38a | *Festuca valesiaca* Gaudin | S | IT-ES | 78 | *Vicia subvillosa* (Ledeb.) Boiss. | SR/CSR | IT |
| 38b | *Festuca valesiaca* Gaudin | S/SR | IT-ES |  |  |  |  |
| 39 | *Gundelia tournefortii* L. | CS | IT |  |  |  |  |

PL: Pluri-regional; ES: Euro-Siberian; M: Mediterranean; IT: Irano-Turanian; KK: the Khorassan-Kopet Dagh floristic province (of the Irano-Turanian region).

*Regional endemic species.

**Iranian endemic species.

**Table S3**. Relationships between CWM-CSR and environmental variables from a Pearson’s correlation coefficient and significance test using linear regressions.

|  | CWM-C | | | CWM-S | | | CWM-R | | |
| --- | --- | --- | --- | --- | --- | --- | --- | --- | --- |
| Variables | **Pearson’s**  **correlation** | **R^2^** | **Pr(>r)** | **Pearson’s**  **correlation** | **R^2^** | **Pr(>r)** | **Pearson’s**  **correlation** | **R^2^** | **Pr(>r)** |
| clay | 0.0668 | 0.0045 | 0.5687 | 0.0246 | 0.0006 | 0.8341 | -0.1270 | 0.0161 | 0.277 |
| silt | -0.0484 | 0.0023 | 0.6799 | 0.1954 | 0.0382 | **0.0929^.^** | -0.2629 | 0.0691 | **0.0227*** |
| sand | 0.1165 | 0.0135 | 0.3195 | -0.2329 | 0.0542 | **0.0443*** | 0.2376 | 0.0565 | **0.0401*** |
| pH | -0.332 | 0.1103 | **0.0036**** | 0.3270 | 0.1070 | 0**.0042**** | -0.1167 | 0.0136 | 0.3188 |
| EC | -0.0378 | 0.0014 | 0.7476 | 0.0527 | 0.0028 | 0.6529 | -0.0390 | 0.0015 | 0.7397 |
| N | -0.0883 | 0.0078 | 0.4513 | 0.0792 | 0.0063 | 0.4994 | 0.10179 | 0.0429 | **0.0745^.^** |
| P | 0.1214 | 0.0147 | 0.2994 | -0.2367 | 0.0561 | **0.0406*** | 0.2380 | 0.0566 | **0.0397*** |
| K | -0.1256 | 0.0157 | 0.2830 | 0.2016 | 0.0406 | **0.0828^.^** | -0.1741 | 0.0303 | 0.1351 |
| org.car | -0.1323 | 0.0175 | 0.2578 | 0.0848 | 0.0072 | 0.4693 | 0.0291 | 0.0008 | 0.8044 |
| org.mat | -0.1193 | 0.0142 | 0.3079 | 0.0677 | 0.0045 | 0.5640 | 0.0409 | 0.0017 | 0.7273 |
| lime | -0.2548 | 0.0649 | **0.0274*** | 0.2947 | 0.0868 | **0.0103*** | -0.1621 | 0.0263 | 0.1648 |
| CEC | -0.0647 | 0.0042 | 0.5813 | 0.0638 | 0.0041 | 0.5866 | -0.0229 | 0.0005 | 0.8455 |
| alt | -0.0187 | 0.0003 | 0.8736 | 0.0778 | 0.0060 | 0.5070 | -0.1057 | 0.0112 | 0.3668 |
| aspect | 0.0302 | 0.0009 | 0.7973 | -0.053 | 0.0028 | 0.6512 | 0.0492 | 0.0024 | 0.6748 |
| bio1 | 0.1124 | 0.0126 | 0.3370 | -0.0937 | 0.0088 | 0.4239 | 0.0112 | 0.00012 | 0.9240 |
| bio2 | 0.0705 | 0.0812 | **0.0132*** | -0.0622 | 0.0492 | **0.0557^.^** | 0.0127 | 5.95e-06 | 0.9834 |
| bio3 | 0.1249 | 0.0639 | **0.0286*** | -0.0928 | 0.0384 | **0.0919^.^** | -0.0065 | 2.234e-07 | 0.9968 |
| bio4 | -0.3049 | 0.01875 | 0.2414 | 0.2064 | 0.0072 | 0.4680 | 0.0493 | 0.0012 | 0.7664 |
| bio5 | -0.0183 | 0.0003 | 0.8764 | -0.0465 | 0.0360 | 0.1030 | -0.0535 | 0.0024 | 0.6760 |
| bio6 | -0.4245 | 0.0027 | 0.6538 | 0.2782 | 8.328e-05 | 0.9381 | 0.0838 | 0.0027 | 0.6547 |
| bio7 | 0.4428 | 0.1150 | **0.0029**** | -0.2795 | 0.0696 | **0.0222*** | -0.1054 | 6.126e-06 | 0.9832 |
| bio8 | 0.1579 | 0.0643 | **0.0282*** | -0.0899 | 0.0213 | 0.2112 | -0.0536 | 0.0070 | 0.4752 |
| bio9 | -0.4192 | 0.0173 | 0.2599 | 0.2697 | 0.0109 | 0.3720 | 0.0910 | 1.866e-05 | 0.9707 |
| bio10 | -0.4306 | 0.0049 | 0.5476 | 0.2785 | 0.0038 | 0.5961 | 0.0911 | 0.0001 | 0.9141 |
| bio11 | 0.3007 | 0.0156 | 0.2857 | -0.1993 | 0.0086 | 0.4285 | -0.0557 | 4.201e-05 | 0.9560 |
| bio12 | 0.2849 | 0.0929 | **0.0078**** | -0.2219 | 0.0426 | **0.0755^.^** | 0.0024 | 0.0024 | 0.6742 |
| bio13 | 0.2529 | 0.0104 | 0.3849 | -0.1960 | 0.0022 | 0.6917 | 0.0005 | 0.0028 | 0.6486 |
| bio14 | -0.1369 | 0.1802 | **0.0001***** | 0.0851 | 0.0774 | 0.0157 | 0.0348 | 0.0070 | 0.4746 |
| bio15 | 0.2072 | 0.1961 | **6.9e-05***** | -0.1897 | 0.0781 | **0.0152*** | 0.0490 | 0.0111 | 0.3681 |
| bio16 | -0.0526 | 0.0249 | 0.1759 | 0.0091 | 0.0081 | 0.4427 | 0.0525 | 0.0029 | 0.6477 |
| bio17 | 0.3391 | 0.1757 | **0.0002***** | -0.2638 | 0.0728 | **0.0192*** | 0.0025 | 0.0083 | 0.4373 |
| bio18 | -0.2536 | 0.1854 | **0.0001***** | 0.14605 | 0.0776 | **0.0155*** | 0.0837 | 0.0083 | 0.4370 |
| bio19 | 0.1317 | 0.0904 | **0.0087**** | -0.1045 | 0.0397 | **0.0864^.^** | 0.0043 | 0.0031 | 0.6351 |

**Table S4.** Environmental variables used in the study. Climate data were obtained from WorldClim ([www.worldclim.org](http://www.worldclim.org)).

| Variable | Abbreviation | Data source |
| --- | --- | --- |
| Annual mean temperature | bio1 | Worldclim |
| Mean diurnal range | bio2 | Worldclim |
| Isothermality | bio3 | Worldclim |
| Temperature seasonality | bio4 | Worldclim |
| Max temperature of warmest month | bio5 | Worldclim |
| Min temperature of coldest month | bio6 | Worldclim |
| Temperature annual range | bio7 | Worldclim |
| Mean temperature of wettest quarter | bio8 | Worldclim |
| Mean temperature of driest quarter | bio9 | Worldclim |
| Mean temperature of warmest quarter | bio10 | Worldclim |
| Mean temperature of coldest quarter | bio11 | Worldclim |
| Annual precipitation | bio12 | Worldclim |
| Precipitation of wettest month | bio13 | Worldclim |
| Precipitation of driest month | bio14 | Worldclim |
| Precipitation seasonality | bio15 | Worldclim |
| Precipitation of wettest quarter | bio16 | Worldclim |
| Precipitation of driest quarter | bio17 | Worldclim |
| Precipitation of warmest quarter | bio18 | Worldclim |
| Precipitation of coldest quarter | bio19 | Worldclim |
| Clay content | clay | calculated |
| Silt content | silt | calculated |
| Sand content | sand | calculated |
| Soil pH | pH | calculated |
| Electrical conductivity of soil (μs/m) | EC | calculated |
| Soil total nitrogen (%) | N | calculated |
| Soil phosphorus (%) | P | calculated |
| Soil potassium (%) | K | calculated |
| Soil organic carbon content | org.car | calculated |
| Soil organic matter content | org.mat | calculated |
| Calcium carbonate (%) | lime | calculated |
| Cation exchange capacity of soil (meq /100 g) | CEC | calculated |
| Elevation (m) | alt | — |
| Aspect (%) | aspect | — |
